# Spinal Tuberculosis Secondary to Intravesical Bacille Calmette-Guerin Treatment for Bladder Cancer

**DOI:** 10.7759/cureus.17446

**Published:** 2021-08-25

**Authors:** Celeste G Yergin, Ryan Pafford, John Pirris, Dinesh Rao, Gazanfar Rahmathulla

**Affiliations:** 1 Neurological Surgery, University of Florida College of Medicine, Jacksonville, USA; 2 Cardiothoracic Surgery, University of Florida College of Medicine, Jacksonville, USA; 3 Neuroradiology, University of Florida Health, Jacksonville, USA

**Keywords:** non-invasive, bladder cancer, intravesical bcg, spinal osteomyelitis, thoracic myelopathy

## Abstract

Intravesical administration of bacille Calmette-Guérin (BCG) is an important component of the gold standard in treating non-muscle invasive bladder cancer (NMIBC). However, complications of this treatment include infections caused by the dissemination of *Mycobacterium bovis*. We present a case of a 62-year-old man who had been treated with intravesical BCG for bladder cancer and developed an *M. bovis* infection of his vertebral column. About four months after completing the BCG treatment, he developed an acute onset of severe upper thoracic radicular back pain, with radiation anteriorly to his sternum. Examination revealed the presence of early myelopathy. After other causes were ruled out, he was diagnosed with the infection four months later. He was investigated for the pain, with resulting imaging identifying an erosive ventral epidural mass at the T4-T5 levels causing cord compression. The patient underwent a transthoracic procedure to evacuate the paraspinal mass lesion and obtain a diagnostic biopsy, followed by a posterolateral decompression of the lesion and posterior instrumented stabilization. Pathology resulted in the identification of a granuloma with a single acid-fast bacillus (AFB) from the paraspinal abscess, thus being diagnostic of a mycobacterial granuloma with paraspinal involvement.

We subsequently performed an extensive review of current literature, looking at articles on spinal osteomyelitis following intravesical BCG treatment of bladder cancer. We identified 26 documented cases in English literature. We present our case report with a good outcome at 24 months, resolving with appropriate chemotherapy. Additionally, we completed a systematic review of the literature and discuss this infrequent iatrogenic pathology. Our report reveals the good response to targeted therapy in the case of osteomyelitis at other skeletal sites and that practitioners caring for these patients maintain a high degree of suspicion in the workup of these patients. Early identification and treatment can appropriately treat osteomyelitis with good long-term outcomes.

## Introduction

Intravesical administration of bacille Calmette-Guérin (BCG) is the treatment of choice for high-risk non-muscle-invasive bladder cancer (NMIBC) and a treatment option for intermediate-risk NMIBC following transurethral resection [1]. BCG treatment comprises an induction phase of six weekly treatments, beginning two to four weeks after resection, and a maintenance course, often with a single BCG dose every three months for one to three years [2,3]. This non-specific immunotherapy was introduced in 1976 [4]. BCG treatment has been shown to reduce tumor recurrence and progression to invasive bladder cancer and regarded as the gold standard for over 40 years. It is an important organ-sparing treatment [2].

Intravesical BCG has been found to cause vertebral osteomyelitis, similar to Pott’s Disease, caused by *Mycobacterium tuberculosis* and observed as early as 1992, requiring the patient to undergo surgical intervention with decompression and anterior lumbar interbody fusion (ALIF) [5]. In addition, other granulomatous infections such as epididymo-orchitis [6] and renal infection [7] have been reported as a result of BCG treatment. We treated a patient with thoracic osteomyelitis secondary to intravesical BCG administration for bladder cancer. There have been other case reports, we have reviewed systemic disease from intravesical BCG in the axial and appendicular skeleton as a treatment for NMIBC, and discussed relevant literature, diagnosis, and treatment outcomes.

## Case presentation

**Figure 1:**
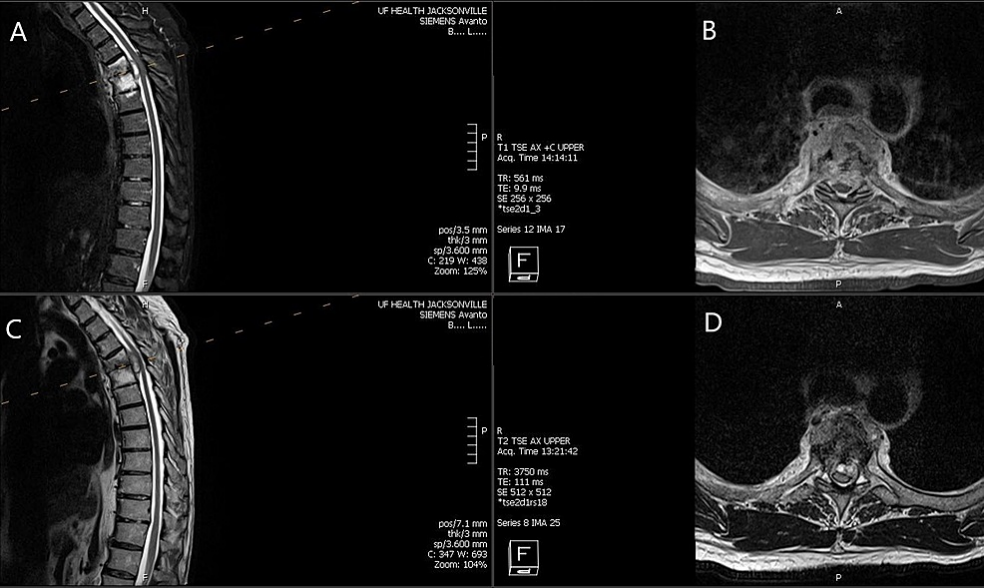
Preoperative MRI sagittal T2 STIR and T2 images (A, C), axial T1 with contrast and T2 (B, D) A: Sagittal T2 short tau inversion recovery (STIR) weighted images of thoracic spine revealing loss of disc space height at T4-T5 with increased T2 signal within the T4 and T5 vertebral bodies, erosion, and involvement of the disc space. There is a well-defined epidural mass lesion causing severe cord compression with cord changes. B: Axial T1-weighted MRI with contrast demonstrating enhancing lesion involving the disc space and adjacent vertebral body with cord compression. There is an abnormal enhancement of the T4 and T5 vertebral bodies with disc space involvement and pre-vertebral collection. C: Sagittal T2 image clearly showing the epidural mass as well as paraspinal involvement, erosion of the disc and the vertebral body with compression of the cord and the lesion which appears epidural. D: Axial T2 images revealing epidural involvement circumferentially more on the left side and ventrally located causing dural/cord compression with involvement of the prevertebral region and hyperintensity within the adjacent rib

A 62-year-old man, with no significant past medical history, was diagnosed with non-invasive bladder cancer in 2016. The patient received six rounds of intravesical BCG treatments and an additional BCG treatment with Interferon-G following the BCG treatments. Subsequently, two follow-up cystoscopic examinations for routine surveillance did not reveal any evidence of cancer recurrence. About five to six months following the negative cystoscopic exams, the patient presented to our hospital emergency department with symptoms of new onset of severe axial back pain along with thoracic radicular symptoms, with radiation of pain to the sternum. Additionally, on examination, the patient had signs of early myelopathy, with numbness about an inch below the nipple line a month prior to presentation at our clinic with exaggerated lower extremity reflexes and increased tone, pathological reflexes. The patient was extensively worked up for alternate causes of chest pain, and the cardiac and upper gastrointestinal exams were negative. Imaging of the thoracic spine was completed because of the symptomatic and clinical localization to the mid-thoracic dermato-myotomes. MRI images (Figure 1 A - D) revealed a contrast-enhancing mass lesion with paraspinal extension, erosion of the adjacent bone and disc space, and ventral lateral epidural extension causing spinal cord compression.

**Figure 2:**
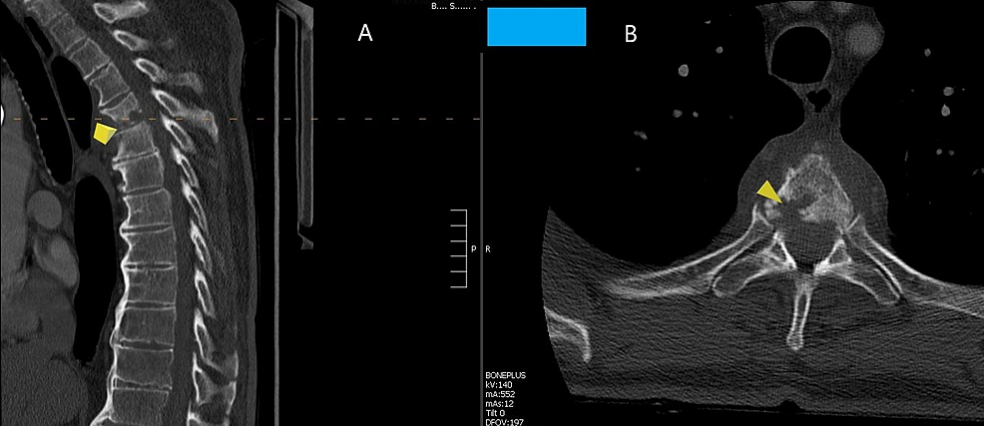
Preoperative CT image sagittal and axial of the thoracic spine A: Sagittal CT image revealing loss of disc height at T4-T5 with erosive changes of the end-plates and the vertebral bodies (marked by yellow arrow) B: axial image revealing erosion and loss of bone in the vertebral body at L4 more on the right side (marked by yellow arrow)

CT of the thoracic spine (Figure 2 A & B) revealed erosive changes within the vertebral bodies and adjacent endplates along with disc height loss.

Based on the imaging, a diagnostic biopsy was performed. The patient underwent a CT-guided bone biopsy with interventional radiology (IR). Preliminary reports were negative for malignancy, and cultures were negative. In order to differentiate between metastatic tumor and infection, we decided to obtain tissue from the paraspinal abscess via a transthoracic approach. Along with cardiothoracic surgery (CTS), the patient underwent video-assisted thoracoscopic surgery (VATS) for aspiration and biopsy of the pre/paravertebral soft tissue abscess and several biopsies of the lesion adjacent to T5 were taken. The clinical appearance was characteristic of a caseous necrotic purulent yellow material. The patient had symptomatic progression of his myelopathy, and we decided to decompress the dural tube and cord, stabilize the patient to relieve the symptoms, and treat him accordingly. While his cultures were awaiting, he underwent a posterolateral T4-T5 decompression with a laminectomy, along with evacuation of a granulomatous mass. This was followed by a T3-T6 posterior thoracic fusion. Intraoperative findings revealed a thick firm, vascular yellow grey tissue mass with necrotic cheesy material surrounding the thecal sac and exiting nerve roots. It was sent for pathology and appropriate cultures. Although several samples were obtained from both these procedures, only one sample was AFB positive.

Based on the intraoperative findings, AFB staining, and pathology of granuloma in consult with infectious disease, he was started on anti-mycobacterial treatment with isoniazid (INH), rifampin, and ethambutol. Subsequently, the patient completed one year of anti-mycobacterial treatment with healing and fusion along with the resolution of his myelopathic symptoms. His follow-up contrasted MRI of the thoracic spine at 18 months post evacuation of spinal mass with instrumented fusion showed resolution of prior T4-T5 vertebral osteomyelitis and stable hardware.

**Figure 3:**
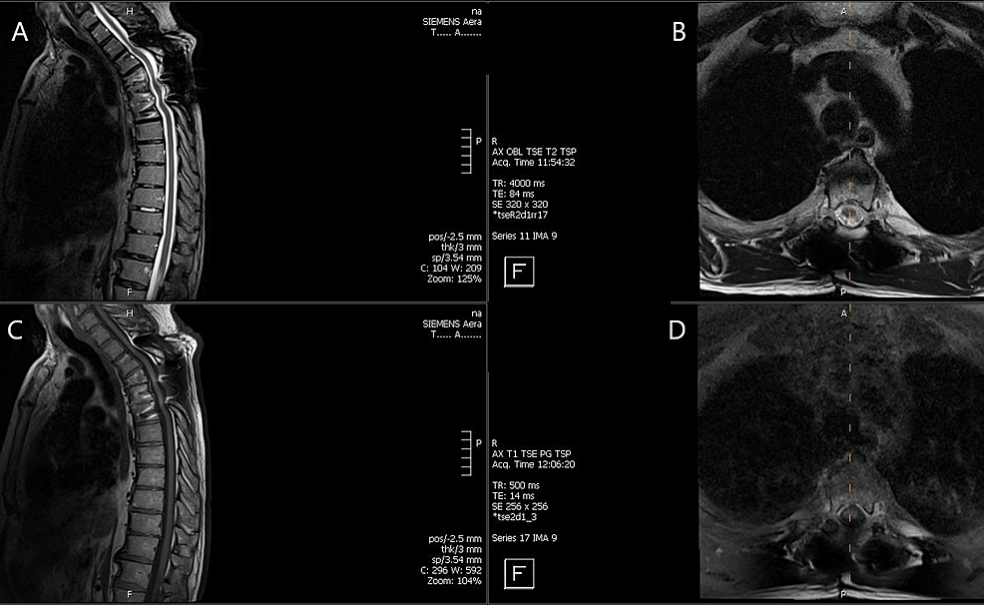
MRI of the thoracic spine 18 months following surgery A and C: Sagittal MRI T2 and T1 without contract revealing complete resolution of the mass lesion with artifact from instrumentation but no mass effect or cord compression B and D: Axial T2 and T1 weighted images with no epidural mass in the T4-T5 region and a resolved prevertebral lesion

At clinical follow up at 18 months post-surgical intervention, the patient was not experiencing back pain, radicular symptoms, numbness, or weakness 

## Discussion

The most common sites of osteomyelitis in adults are the vertebrae, with *Staphylococcus aureus* being the most common cause and b-hemolytic *Streptococcus* species, *Haemophilus influenzae*, *Kingella kingae*, *Brucella* species, and *Mycobacterium tuberculosis* as other etiologies [8,9]. Destruction of the vertebral body can cause spinal column instability and spinal cord damage, resulting in neurological symptoms. Of the patients with vertebral osteomyelitis, 50% are treated medically with appropriate antibiotics [10], while the others undergo surgical intervention, with neurological deficits, sepsis, spinal instability, and failure of medical management as the main indications for surgery [8]. Issa et al. reported that in 2013, there were 16,917 cases of vertebral osteomyelitis admissions, with a mortality rate of 2.2% during hospital stay, and a mean estimated hospital charge of $80,786 [11], showing that iatrogenic vertebral osteomyelitis would be a huge cost that is best avoided.

As we have found, our 62-year-old patient is not rare in developing *M. bovis* osteomyelitis of the vertebrae spinal column after intravesical BCG treatment for bladder cancer. Bladder cancer accounts for approximately 500,000 cases of bladder cancer worldwide and over 80,000 cases in the US alone each year, and NMIBC accounts for over 70% of these cases [1,2]. Intravesical BCG is the gold-standard treatment for patients with NMIBC following transurethral resection - one review states that 28% of patients with intermediate-risk NMIBC receive BCG treatment of some sort [12] and another states that most patients manifesting squamous or glandular subtypes of high-grade NMIBC, and who do not undergo radical cystectomy, benefit from BCG treatments and do receive the treatment [13]. Despite so many patients receiving intravesical BCG, the incidence of this iatrogenic infection is extremely low. This suggests that in the absence of predisposing conditions, immunosuppression, for example, the risk of developing *M. bovis* osteomyelitis is very low.

In terms of the efficacy of intravesical BCG as a treatment for bladder cancer, 56% of patients had their tumors eradicated as a result of the treatments [14,15]. In addition, studies have shown that patients who received maintenance therapy in addition to induction therapy had a significantly lower risk of disease progression [16,17]. Martínez-Piñeiro et al. [18] agree that patients who receive maintenance BCG therapy for three years after the induction therapy had lower cancer recurrence and progression, but also report that there are more side effects resulting from BCG therapy. They also report that patients who received a modified maintenance therapy of a single BCG installation every three months for three years did not perform better than patients who receive only induction therapy. This suggests that the typical schedule of additional maintenance BCG treatments does afford certain patients better prognoses. The current mindset is at least one year of maintenance BCG given once a week for three weeks at three, six, and 12 months after initial BCG treatment, and three years in patients with a higher risk of cancer recurrence [19]. In contrast, patients with a lower grade (TaG1-G2) bladder cancer, do not benefit from additional maintenance therapy [20]. Ishawal et al. [21] reported that one of the most prognostic factors for bladder cancer progression is the patient’s response to BCG treatment at the three- or six-month follow-up, in addition to other factors like the grade of tumor, size, and depth of lamina propria invasion. As such, it may be prudent to determine if patients require maintenance therapy in addition to induction therapy and if so, are they able to receive and tolerate both courses, before initiating BCG treatment.

Of course, in addition to bony infections, several other infections resulting from BCG treatment have been reported [22] in patients who have been treated BCG and present with unexplained symptoms. Even though these infections typically do not cause as severe neurologic damage as osteomyelitis, *M. bovis* infection should be always on their physician’s differential diagnosis when working up these patients. In addition, serious complications of this treatment such as infection of various organ systems and causing inflammatory processes have been documented [3].

One interesting finding is that with the addition to our patient, all the reports of *M. bovis* osteomyelitis resulting from intravesical BCG treatment for bladder cancer are all in men. The incidence of bladder cancer in men is only three to four times higher than in women [2]. Women who are diagnosed with muscle-invasive bladder cancer will not benefit from BCG treatment, and along with reports that women do not respond as well to BCG treatment [23], fewer women actually receive BCG installations for bladder cancer, and thus the absence of reports of *M. bovis* osteomyelitis in women.

## Conclusions

Spinal osteomyelitis is a rare occurrence after BCG installation. The benefits of BCG treatment for NMIBC outweigh any iatrogenic medical risks, as their occurrence is infrequent. Early diagnosis and treatment medically, surgically, or in combination have a good prognosis and functional outcome for these patients.

## References

[1] Golla V, Lenis AT, Faiena I, Chamie K (2019). Intravesical therapy for non-muscle invasive bladder cancer—current and future options in the age of bacillus Calmette-Guerin shortage. Rev Urol.

[2] Lenis AT, Lec PM, Chamie K, Mshs MD (2020). Bladder cancer: a review. JAMA.

[3] Alhunaidi O, Zlotta AR (2019). The use of intravesical BCG in urothelial carcinoma of the bladder. Ecancermedicalscience.

[4] Morales A, Eidinger D, Bruce AW (1976). Intracavitary bacillus Calmette-Guerin in the treatment of superficial bladder tumors. J Urol.

[5] Katz DS, Wogalter H, D’esposito RF, Cunha BA (1992). Mycobacterium bovis vertebral osteomyelitis and psoas abscess after intravesical BCG therapy for bladder carcinoma. Urology.

[6] Parker SG, Kommu SS (2013). Post-intravesical BCG epididymo-orchitis: case report and a review of the literature. Int J Surg Case Rep.

[7] Bajramovic S, Alic J, Skopljak E, Chikha A, Vesnic S, Smajilbegovic V, Aganovic D (2020). Renal tuberculosis following intravesical bacillus Calmette-Guérin (BCG) immunotherapy for the treatment of Bbadder cancer. Med Arch.

[8] Boody BS, Tarazona DA, Vaccaro AR (2018). Evaluation and management of pyogenic and tubercular spine infections. Curr Rev Musculoskelet Med.

[9] Colston J, Atkins B (2018). Bone and joint infection. Clin Med (Lond).

[10] Cornett CA, Vincent SA, Crow J, Hewlett A (2016). Bacterial spine infections in adults: evaluation and management. J Am Acad Orthop Surg.

[11] Issa K, Diebo BG, Faloon M, Naziri Q, Pourtaheri S, Paulino CB, Emami A (2018). The epidemiology of vertebral osteomyelitis in the United States from 1998 to 2013. Clin Spine Surg.

[12] Kamat AM, Witjes JA, Brausi M (2014). Defining and treating the spectrum of intermediate risk nonmuscle invasive bladder cancer. J Urol.

[13] Suh J, Moon KC, Jung JH (2019). BCG instillation versus radical cystectomy for high-risk NMIBC with squamous/glandular histologic variants. Sci Rep.

[14] Haaff EO, Dresner SM, Ratliff TL, Catalona WJ (1986). Two courses of intravesical bacillus Calmette-Guerin for transitional cell carcinoma of the bladder. J Urol.

[15] Steinberg RL, Thomas LJ, O'Donnell MA (2015). Bacillus Calmette-Guérin (BCG) treatment failures in non-muscle invasive bladder cancer: what truly constitutes unresponsive disease. Bladder Cancer.

[16] Hinotsu S, Akaza H, Naito S (2011). Maintenance therapy with bacillus Calmette-Guérin Connaught strain clearly prolongs recurrence-free survival following transurethral resection of bladder tumour for non-muscle-invasive bladder cancer. BJU Int.

[17] Sylvester RJ, van der MEIJDEN AP, Lamm DL (2002). Intravesical bacillus Calmette-Guerin reduces the risk of progression in patients with superficial bladder cancer: a meta-analysis of the published results of randomized clinical trials. J Urol.

[18] Martínez-Piñeiro L, Portillo JA, Fernández JM (2015). Maintenance therapy with 3-monthly bacillus Calmette-Guérin for 3 years is not superior to standard induction therapy in high-risk non-muscle-invasive urothelial bladder carcinoma: final results of randomised CUETO Study 98013. Eur Urol.

[19] (2021). UpToDate: Patient education: bladder cancer treatment; non-muscle invasive (superficial) cancer (beyond the basics). https://www.uptodate.com/contents/bladder-cancer-treatment-non-muscle-invasive-superficial-cancer-beyond-the-basics.

[20] Andius P, Holmäng S (2004). Bacillus Calmette-Guérin therapy in stage Ta/T1 bladder cancer: prognostic factors for time to recurrence and progression. BJU Int.

[21] Isharwal S, Konety B (2015). Non-muscle invasive bladder cancer risk stratification. Indian J Urol.

[22] Green DB, Kawashima A, Menias CO (2019). Complications of intravesical BCG immunotherapy for bladder cancer. Radiographics.

[23] Palou J, Sylvester RJ, Faba OR, Parada R, Peña JA, Algaba F, Villavicencio H (2012). Female gender and carcinoma in situ in the prostatic urethra are prognostic factors for recurrence, progression, and disease-specific mortality in T1G3 bladder cancer patients treated with bacillus Calmette-Guérin. Eur Urol.

